# Systemic endothelial activation and eosinophilic inflammation in pediatric allergic rhinitis: diagnostic value of endocan and eosinophil-derived neurotoxin

**DOI:** 10.1007/s00431-026-06975-7

**Published:** 2026-04-30

**Authors:** Handan Ayhan Akoğlu, Hilmi Furkan Arslan, Mahmut Doğru

**Affiliations:** 1 Department of Pediatrics, Giresun University Faculty of Medicine, Giresun, Turkey; 2 Department of Medical Biochemistry, Giresun University Faculty of Medicine, Giresun, Turkey; 3 Department of Pediatric Allergy and Immunology, Memorial Şişli Hospital, Istanbul, Turkey

**Keywords:** Allergic rhinitis, Endocan, Eosinophil-derived neurotoxin, Endothelial activation, Eosinophilic inflammation

## Abstract

**Abstract:**

Objective biomarkers that reflect systemic inflammation in pediatric allergic rhinitis to support clinical diagnosis. We aimed to evaluate serum levels of endocan (a marker of endothelial activation) and eosinophil-derived neurotoxin (EDN; reflecting eosinophil degranulation) in children with AR and investigate their diagnostic performance and associations with disease severity and conventional inflammatory markers. In this prospective case-control study, 85 children with AR and 67 healthy controls were enrolled. Serum endocan and EDN were measured via sandwich ELISA. Primary outcomes included group comparisons of biomarker levels and their diagnostic accuracy determined by receiver operating characteristic (ROC) curve analysis. Serum endocan and EDN levels were significantly elevated in children with AR compared to controls (both *p* < 0.001). Both biomarkers demonstrated high diagnostic performance, with an area under the curve (AUC) of 0.931 for endocan and 0.929 for EDN, showing greater diagnostic accuracy than absolute eosinophil counts (AUC, 0.871). Endocan and EDN showed a strong intercorrelation (r = 0.88, *p* < 0.001) and significant positive correlations with total IgE and eosinophil counts. However, no significant associations were observed between these biomarkers and disease severity, symptom control scores, or allergen sensitization patterns.

*Conclusions*: Serum endocan and EDN are significantly elevated in children with AR and demonstrate high diagnostic discrimination in this cohort. These findings suggest that endocan and EDN may serve as promising complementary biomarkers for the objective assessment of allergic inflammation in children, although further multicenter studies are needed to confirm their clinical utility.

**Table:** 

**What's Known:** • *Allergic rhinitis (AR) is common in childhood and involves systemic inflammatory pathways; however, objective biomarkers to support diagnosis in pediatric practice remain limited.* • *Endocan and eosinophil-derived neurotoxin (EDN) reflect endothelial activation and eosinophil degranulation, respectively, and have been studied in other atopic and inflammatory conditions.* **What is New:** • *This study concurrently evaluates serum Endocan and EDN in children with AR in a prospective case–control design.* •* Both biomarkers demonstrated high discriminatory performance (AUC∼ 0.93 in this cohort) and showed greater diagnostic accuracy than absolute eosinophil counts .* • *Endocan and EDN are largely independent of generalized systemic inflammatory indices (NLR, SII, SIRI), supporting specificity for the endothelial— eosinophilic axis in pediatric AR.*

**Graphical abstract:**

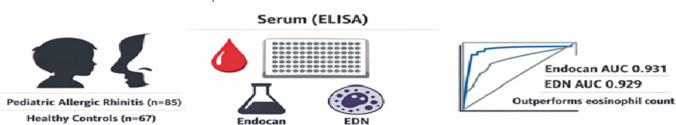

## Introduction

Allergic rhinitis (AR) is a chronic inflammatory disease of the nasal mucosa characterized by IgE-mediated responses to aeroallergens, such as pollens, house dust mites, and animal dander. Clinically manifested by rhinorrhea, sneezing, and nasal and ocular pruritus, AR affects a significant proportion of the pediatric population worldwide [1].

Endocan (endothelial cell-specific molecule-1) is a dermatan sulfate proteoglycan primarily expressed by vascular endothelial cells in the lungs and kidneys [2, 3]. It plays a pivotal role in pro-inflammatory responses by upregulating adhesion molecules, reorganizing the endothelial cytoskeleton, and interacting with the NF-кB signaling pathway, thereby serving as a recognized marker of endothelial activation [3]. Elevated endocan levels have been implicated in various allergic conditions, including asthma, atopic dermatitis, and hereditary angioedema, with studies suggesting a correlation between its concentration and disease severity [4–7]. Notably, children with asthma exhibit higher serum endocan levels compared to healthy controls, often proportional to airway obstruction severity [4]. Similarly, increased nasal endocan levels have been reported in pediatric AR [8], suggesting that endocan may serve as a systemic or local indicator of atopic sensitization and chronic allergic inflammation [4–8].

Eosinophil-derived neurotoxin (EDN), a granule protein released upon eosinophil activation, has emerged as a significant biomarker for pediatric allergic airway diseases [9]. High EDN levels in early childhood are associated with an increased risk of developing allergic diseases within the first 3 years of life [10]. Considering the concept of the “atopic march”—which describes a common clinical trajectory in many allergic individuals from atopic dermatitis and food allergy to asthma and AR—identifying biomarkers that reflect this potential progression is of clinical importance, and EDN has been proposed as a promising candidate in this context. Specifically, EDN levels rise significantly in allergic airway diseases, particularly after the age of three [9–11].

While the predictive value of EDN for asthma presence and severity is well-documented, its role in AR remains less explored [4, 9, 12]. Notably, among various eosinophilic indices (including serum eosinophil cationic protein and absolute eosinophil count (AEC)), EDN has been shown to significantly differentiate between asthma severity levels during acute exacerbations [9], suggesting that EDN may be a more sensitive indicator of disease activity than conventional eosinophilic markers. However, the clinical utility of concurrently evaluating endothelial activation and eosinophil degranulation in pediatric AR has not been systematically investigated. Therefore, we aimed to evaluate serum Endocan and EDN levels in children with AR and investigate their associations with disease severity, symptom scores, and inflammatory indices to clarify their potential diagnostic utility.

## Materials and methods

### Study design and population

This prospective case–control study was conducted between March 2024 and March 2025 at the pediatric outpatient clinic of Giresun University Training and Research Hospital, a tertiary care center.

The AR group consisted of patients aged < 18 years diagnosed with AR. Patients were classified according to the Allergic Rhinitis and its Impact on Asthma (ARIA) guidelines [13] into four categories: mild–intermittent, moderate–to–severe intermittent, mild–persistent, and moderate–to–severe persistent. The Control group comprised age-matched healthy volunteers aged less than 18 years who presented for routine health screening and had no history of allergic or chronic diseases.

Exclusion criteria for both groups included age > 18 years, presence of chronic conditions (e.g., diabetes mellitus, chronic kidney disease, and cardiac or neurological disorders), comorbid allergic diseases (asthma and atopic dermatitis), and regular use of any systemic medications. The exclusion of comorbidities was intended to isolate the specific association of the investigated biomarkers with AR.

### Sample size calculation

The sample size was determined based on a previous study comparing eosinophil-derived neurotoxin levels in children with asthma [12]. Based on an assumed effect size of 0.80, a 5% type 1 error rate, and 80% power, the minimum required sample size was calculated as 40 participants per group.

### Data collection and inflammatory indices

Sociodemographic characteristics, atopic status, and family history of allergy were recorded. For the AR group, disease severity was classified according to ARIA criteria, and symptom control was assessed using the Rhinitis Control Assessment Test (RCAT). Laboratory data, including complete blood count (CBC) parameters, AEC, total IgE levels, and skin prick test (SPT) results, were retrieved from medical records.

Systemic inflammatory indices were calculated from CBC parameters obtained at enrollment as follows:

- Neutrophil-to-lymphocyte ratio (NLR): neutrophil count/lymphocyte count,
- Platelet-to-lymphocyte ratio (PLR): platelet count/lymphocyte count,
- Lymphocyte-to-monocyte ratio (LMR): lymphocyte count/monocyte count.
- Systemic Immune-Inflammation Index (SII): (platelet count × neutrophil count)/lymphocyte count
- Systemic Inflammation Response Index (SIRI): (neutrophil count × monocyte count)/lymphocyte count.

### Sample collection and biochemical analysis

To ensure standardized conditions and minimize variability in biomarker levels, venous blood samples were obtained in the morning following a 10–12 h fast. To obtain the serum, samples were centrifuged at 1500 × g for 15 min immediately after collection. The supernatant was then aliquoted into Eppendorf tubes and stored at − 80 °C until analysis. Prior to analysis, serum samples were gradually thawed to room temperature. Serum endocan and EDN concentrations were quantified using commercially available in vitro sandwich ELISA kits (Elabscience, Texas, USA) according to the manufacturer’s instructions. Absorbance measurements for serum EDN and endocan levels were performed using a microplate reader (AccuReader, Metertech Inc., Taipei, Taiwan).

### Statistical analysis

Data analysis was performed using SPSS version 26.0 (IBM Corp., Armonk, NY, USA). Descriptive statistics were presented as mean ± standard deviation (SD) for normally distributed variables, median (25th–75th percentiles) for non-normally distributed variables, and frequencies (percentages) for categorical variables. The normality of data distribution was assessed using the Kolmogorov–Smirnov test. Differences in serum endocan, EDN, and inflammatory indices between the AR and control groups were analyzed using the independent samples *t*-test or the Mann–Whitney *U* test, as appropriate. Categorical variables were compared using the chi-square test. Correlations between quantitative variables were evaluated using Pearson or Spearman correlation coefficients. To account for multiple comparisons in the correlation analyses between serum biomarkers and systemic inflammatory indices (NLR, PLR, LMR, SII, and SIRI), *p*-values were adjusted using the Benjamini–Hochberg false discovery rate (FDR) procedure with a *q*-value of 0.05. Receiver operating characteristic (ROC) curve analysis was performed to determine the diagnostic performance of absolute eosinophil counts, endocan, and EDN. A *p*-value of < 0.05 was considered statistically significant.

## Results

A total of 152 children were included: 85 with allergic rhinitis (AR) and 67 healthy controls. The mean age was 8.5 ± 3.1 years (median 7, range 4–17) in the AR group and 9.7 ± 3.7 years (median 10, range 4–17) in the control group (*p* > 0.05). Among children with AR, 56% were first-born and 84% were born at term. A family history of allergy was reported in mothers (31%), fathers (22%), and siblings (40%); the prevalence of AR among family members was 13% in mothers, 8.4% in fathers, and 15.9% in siblings. The most common AR symptom was nasal obstruction (91%), followed by sneezing (89%), rhinorrhea (86.7%), nasal/palatal pruritus (77%), and ocular watering (67.5%). Household passive smoke exposure was reported for 53% of the AR group. Skin prick testing revealed polysensitization as the most common pattern (44%), with house dust mites being the leading allergen among monosensitized patients (10%).

Serum EDN, endocan, and absolute eosinophil counts (AEC) were significantly higher in the AR group compared to controls (all *p* < 0.001; Table 1). No significant differences were observed regarding systemic inflammatory indices (NLR, PLR, LMR, SII, SIRI).

**Table 1:** Demographic characteristics, serum biomarker levels, eosinophil counts, and systemic inflammatory indices in children with allergic rhinitis and healthy controls

Variables (median, 25th–75th percentiles)	AR (*n* = 85)	Control (*n* = 67)	*p*
Age (year)	7 (6–10)	10 (6–13)	0.06^a^
Gender (*n* (%))			0.29^b^
Female	51 (50.8%)	35 (49.2%)	
Male	33 (59.3%)	32 (40.7%)	
EDN (ng/mL)	80.8 (59.4–116.9)	37.7 (28.5–49.1)	**< 0.001**^a*^
Endocan (pg/mL), (mean ± sd)	1074.9 ± 347.6	550.8 ± 165	**< 0.001**^c*^
Eosinophil (cells/μL)	590 (255–810)	150 (102–240)	**< 0.001**^a*^
NLR	1.3 (0.9–1.9)	1.3 (1.0–1.8)	0.72^a^
PLR	107.8 (77.2–123.7)	112.1 (88.5–138.1)	0.12^a^
LMR	5.8 (4.4–7.6)	6 (4.9–7.2)	0.55^a^
SII	400,045 (268,609–613,648)	397,427 (307,200–604,632)	0.96^a^
SIRI	672.5 (471.5–1159.1)	551.8 (445.9–859.3)	0.16^a^

Continuous variables are presented as mean ± SD (normally distributed) or median (25^th^–75^th ^percentiles) (non−normally distributed) and categorical variables as *n* (%). Between group comparisons were performed using the independent samples *t*−test (normally distributed continuous variables), Mann–Whitney *U *test (non−normally distributed continuous variables), and Pearson’s chi−square test (categorical variables). Two−tailed tests were used; *p* < 0.05 was considered statistically significant

*EDN* eosinophil-derived neurotoxin, *NLR *neutrophil-to-lymphocyte ratio, *PLR* platelet-to-lymphocyte ratio, *LMR *lymphocyte-to-monocyte ratio, *SII* Systemic Inflammation Index, *SIRI *Systemic Inflammation Response Index

^a^Mann-Whitney *U *test

^b^Pearson chi-square test

^c^Independent sample *t* test

^*^p < 0.05

Correlation analyses revealed that serum endocan and EDN were significantly correlated with absolute eosinophil counts and total IgE levels (Table 2). Regarding systemic inflammatory indices, although nominal *p*-values suggested weak correlations between biomarkers and certain indices (PLR, LMR, and SIRI), only the associations between endocan and LMR (*r* =  − 0.24, *p* = 0.004) and EDN and LMR (*r* =  − 0.22, *p* = 0.01) remained statistically significant after Benjamini–Hochberg FDR correction. No significant correlations were found with RCAT scores, passive smoke exposure, allergen sensitization patterns (mono- vs. polysensitization), or ARIA-defined disease severity.

**Table 2:** Correlation of serum EDN and endocan with total IgE, eosinophil counts, systemic inflammatory indices, and RCAT scores

	EDN		Endocan	
	*p*	*r*	*p*	*r*
Total IgE (*n* = 85)	< 0.001^**^	0.44^**^	< 0.001^**^	0.46^**^
Eosinophil (*n* = 145)	< 0.001^**^	0.75^**^	< 0.001^**^	0.76^**^
NLR (*n* = 145)	0.9	0.01	0.76	− 0.25
PLR (*n* = 145)	0.07	− 0.15	0.02	− 0.19
LMR (*n* = 145)	0.01^*^	− 0.22^*^	0.004^*^	− 0.24^*^
SII (*n* = 145)	0.97	− 0.003	0.65	− 0.04
SIRI (*n* = 145)	0.24	0.19	0.03	0.18
RCAT (*n* = 85)	0.54	− 0.07	0.93	− 0.009^a^
Vitamin D (*n* = 145)	0.42	− 0.07	0.25	− 0.09
Ferritin (*n* = 145)	0.25	0.09	0.65	0.04

Correlations were assessed using Spearman’s rank correlation for non−normally distributed variables and Pearson’s correlation for normally distributed variables. Two−tailed *p*−values are presented. *p*−values for correlations with systemic inflammatory indices (NLR, PLR, LMR, SII, SIRI) were adjusted for multiple comparisons using the Benjamini–Hochberg false discovery rate (FDR) procedure. Values marked with an asterisk (*) remained statistically significant after FDR correction (*q*< 0.05)

*EDN* eosinophil-derived neurotoxin, *RCAT* Rhinitis Control Assessment Test, *NLR* neutrophil-to-lymphocyte ratio, *PLR* platelet-to-lymphocyte ratio, *LMR* lymphocyte-to-monocyte ratio, *SII* Systemic Inflammation Index, *SIRI* Systemic Inflammation Response Index

^*^q < 0.05

^**^p < 0.01

^a^Pearson’s correlation

**Fig. 1:**
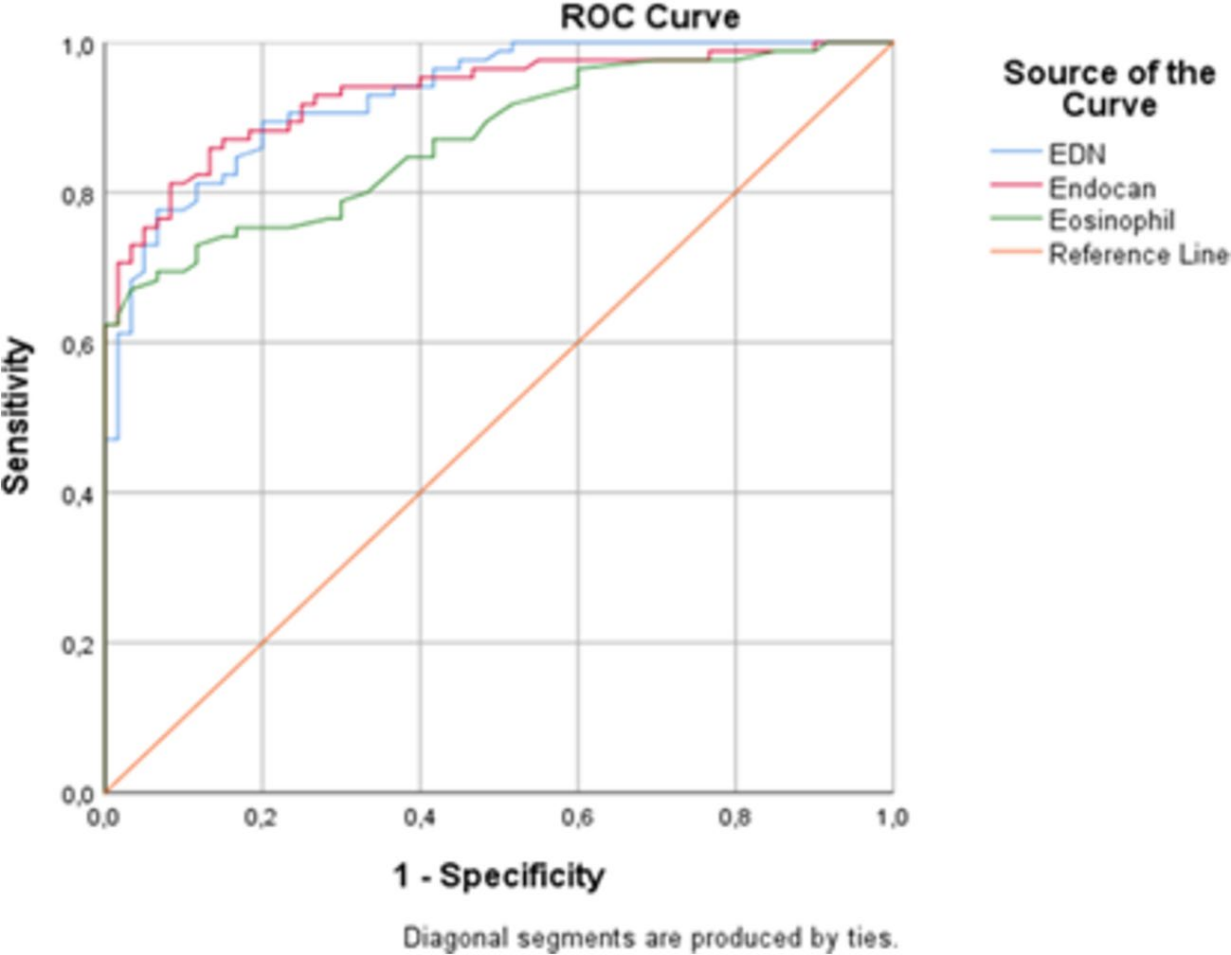
Receiver operating characteristic (ROC) curves showing the diagnostic performance of serum EDN, endocan, and absolute eosinophil counts for allergic rhinitis. The AUC was 0.929 (95% CI 0.891–0.968) for EDN and 0.931 (95% CI 0.893–0.971) for endocan. At the optimal cut-off values (EDN, 50.77 ng/mL; endocan, 748.2 ng/mL), sensitivity and specificity were 89.4%/82.1% and 85.9/86.7%, respectively. Absolute eosinophil count yielded an AUC of 0.871 (95% CI 0.816–0.927); at a cut-off of 455 cells/µL, sensitivity and specificity were 70.2% and 93.3%, respectively. The dashed line indicates chance discrimination (AUC = 0.5). All AUC values were statistically significant (*p* < 0.001)

In ROC curve analysis, endocan yielded an AUC value of 0.931 (95% CI 0.893–0.971), with 85.9% sensitivity and 86.7% specificity at a cut-off of 748.2 pg/mL. EDN yielded an AUC of 0.929 (95% CI 0.891–0.968), with 89.4% sensitivity and 82.1% specificity at a cut-off of 50.77 ng/mL. Both biomarkers showed higher AUC values compared with AEC, which had an AUC of 0.871 (95% CI 0.816–0.927), with 70.2% sensitivity and 93.3% specificity at a cut-off value of 455 cells/μL (all *p* < 0.001) (Fig. 1, Table 3).

**Table 3:** Receiver operating characteristic analysis of endocan and EDN scores in predicting allergic rhinitis

	AUC*	95% confidence interval		Cut-off value	Sensitivity	Specificity	*p*-value
		Lower	Upper				
Endocan	0.931	(0.893–0.971)		748.2 pg/mL	85.9	86.7	< 0.001
EDN	0.929	(0.891–0.968)		50.77 ng/mL	89.4	82.1	< 0.001
Eosinophil	0.871	(0.816–0.927)		455 cells/μL	70.2	93.3	< 0.001

*AUC, area under the ROC curve

## Discussion

In this prospective, case–control study, we compared serum endocan and EDN levels between children with AR and healthy controls and examined their associations with classical markers of allergic inflammation. Our principal findings can be summarized as follows: (i) serum endocan, EDN, and AEC were significantly higher in children with AR; (ii) endocan and EDN showed a strong positive correlation with each other and moderate-to-strong correlations with total IgE and AEC; and (iii) both biomarkers demonstrated high diagnostic performance, showing greater diagnostic accuracy than AEC in ROC analysis. In contrast, neither biomarker correlated with systemic inflammatory indices, RCAT scores, or AR severity.

Serum endocan levels were markedly elevated in children with AR, with high discriminatory ability (AUC 0.931). This finding suggests that the inflammatory response in pediatric AR, traditionally considered localized to the nasal mucosa, may also involve systemic endothelial activation. Although elevated endocan levels have been reported in various inflammatory and allergic conditions [4, 14–18], studies investigating endocan in pediatric AR remain scarce. To our knowledge, only one study has reported elevated nasal endocan levels in children with AR [8]. Our results therefore provide novel evidence supporting the potential role of serum endocan as a possible systemic biomarker of endothelial activation in pediatric AR. However, it should be noted that endocan is not disease-specific and may be elevated in other inflammatory conditions; therefore, its interpretation should be made in the appropriate clinical context.

Similarly, serum EDN levels were significantly elevated and demonstrated high diagnostic accuracy (AUC 0.929). Because EDN reflects that eosinophil activation rather than simply eosinophil number, its elevation indicates active eosinophilic degranulation in AR. Previous researchs have shown that EDN correlates with asthma severity and increases in allergic conditions [9, 11]. However, to our knowledge, no prior study has concurrently evaluated both endocan and EDN in pediatric AR. Our findings support the potential utility of serum EDN as a measurable systemic marker of eosinophilic activation in pediatric AR.

The strong positive correlation between endocan and EDN (*r* = 0.88) and their moderate-to-strong correlations with total IgE and AEC are particularly noteworthy. These results suggest that endothelial activation and eosinophilic inflammation are closely interrelated in pediatric AR and may represent components of a shared inflammatory pathway. As IgE-mediated allergic responses and eosinophil recruitment intensify, endothelial cell activation may also increase, leading to elevated serum endocan levels. This interpretation aligns with contemporary pathophysiological models proposing that AR is not merely a localized nasal disease but may also include systemic inflammatory and vascular components [1, 13].

Notably, after adjusting for multiple comparisons, endocan and EDN remained largely independent of most systemic inflammatory indices, with the exception of a weak correlation with LMR. This lack of robust association with general markers such as NLR or SII underscores that endocan and EDN are not merely surrogates for generalized systemic inflammation but rather may serve as specific indicators of the endothelial—eosinophilic axis in AR. These findings suggest that these biomarkers may reflect underlying chronic disease processes rather than acute symptom fluctuations. This is further supported by the lack of association with RCAT scores, implying that endocan and EDN may serve as more sensitive indicators of disease presence than day-to-day symptom control.

Furthermore, the lack of significant differences by allergen sensitization pattern or AR severity categories suggests that, unlike in pediatric asthma [7, 9], these biomarkers may have limited sensitivity for stratifying disease severity in AR, despite their high performance in distinguishing affected children from healthy individuals. The lack of correlation with ARIA severity suggests that endocan and EDN may reach a “plateau” effect once the threshold for systemic allergic sensitization is crossed, suggesting they may have greater potential as diagnostic adjuncts rather than tools for severity stratification compared to their role in asthma. Similarly, the absence of significant effects of passive smoke exposure suggests that these biomarkers may be relatively independent of the extrinsic inflammatory burden caused by environmental tobacco smoke, although further studies evaluating dose–response relationships are warranted.

One of the most clinically relevant findings of our study is the potential utility of both endocan and EDN as complementary biomarkers in the diagnostic evaluation of pediatric AR. The high AUC values and balanced sensitivity/specificity ratios indicate that these markers may meaningfully contribute to distinguishing children with AR from healthy controls. Although AEC also demonstrated good discriminatory power (AUC 0.871), the fact that endocan and EDN reflect functional aspects of eosinophilic inflammation and endothelial activation renders them valuable beyond conventional hematologic parameters. Nevertheless, it is important to emphasize these biomarkers are not intended to replace guideline-recommended diagnostic tools (e.g., clinical assessment, skin prick testing, and specific IgE measurement) but rather to complement them.

Our findings contribute to the understanding of AR pathophysiology by supporting the concurrent involvement of endothelial activation and eosinophilic inflammation in pediatric AR. Elevated endocan levels suggest that endothelial cells play an active role in mediating leukocyte adhesion and vascular permeability changes during allergic inflammation, while elevated EDN levels point to mechanisms involving eosinophil-mediated tissue damage and potential effects on neuronal structures that may contribute to symptom generation. A deeper understanding of these mechanisms may inform the development of targeted therapeutic strategies in the future.

## Strengths and limitations

Our study has several strengths. The prospective, case–control design enhaces the reliability of our findings. The inclusion of age-matched healthy controls minimizes potential confounding by age. A key strength is the concurrent evaluation of both endocan and EDN in the same cohort providing a unique opportunity to explore the interplay between endothelial activation and eosinophilic inflammation. Furthermore, the inclusion of novel systemic inflammatory indices (SII, SIRI) and the application of rigorous statistical adjustments for multiple comparisons (FDR correction) strengthen the methodological quality of our analysis. To our knowledge, this is the first study to simultaneously investigate these two biomarkers in pediatric AR, representing a novel contribution to the literature.

However, several limitations should be acknowledged. The single-center design may limit the generalizability of our findings to other geographic and ethnic populations. Although our sample size was statistically adequate for the primary comparisons, it may have been insufficient for certain subgroup analyses, such as those based on specific allergen profiles or comorbidities. In addition, children with comorbid allergic conditions (e.g., asthma and atopic dermatitis) were excluded to isolate the effects of AR; however, this may limit the applicability of our findings to real-world clinical settings where such comorbidities are common. In addition, children with comorbid allergic conditions (e.g., asthma and atopic dermatitis) were excluded to isolate the effects of AR; however, this may limit the applicability of our findings to real-world clinical settings where such comorbidities are common.

Additionally, biomarker measurements were performed at a single time point and were not designed to assess treatment response or temporal variability. Serial measurements would be valuable to better understand fluctuations in biomarker levels during disease exacerbations and in response to therapy.

It should also be noted that endocan is not disease-specific and may be elevated in a variety of inflammatory conditions, which may limit its specificity as a standalone biomarker.

Furthermore, although all samples were collected and processed under standardized conditions, potential variability related to pre-analytical and analytical factors (e.g., sample handling and ELISA methodology) cannot be entirely excluded.

Despite these limitations, our study provides valuable insights into the associations of endocan and EDN with each other and with classical allergic parameters in pediatric AR.

Future research should focus on validating endocan and EDN levels in larger, multicenter pediatric cohorts and exploring their associations with AR phenotypes. Evaluating serial measurements before and after pharmacological treatment or allergen immunotherapy will be crucial to assess their predictive value for therapeutic response. Furthermore, long-term follow-up studies are needed to investigate the prognostic implications of these biomarkers on disease trajectory and their potential role in personalized management strategies.

## Conclusion

In conclusion, serum endocan and EDN levels are significantly elevated in children with allergic rhinitis compared to healthy controls in this cohort. These biomarkers show strong correlations with total IgE, AEC, and each other, suggesting the concurrent involvement of systemic endothelial activation and eosinophilic inflammation in pediatric AR. While the high AUC values observed suggest potential adjunctive diagnostic value, these findings should be interpreted with caution given the single-center, cross-sectional design.

Larger multicenter prospective studies are warranted to confirm these findings and define their clinical utility before routine implementation.

## Data Availability

The datasets generated during and/or analyzed during the current study are available from the corresponding author on reasonable request.

## Abbreviations

AR: Allergic rhinitis
AEC: Absolute eosinophil count
ARIA: Allergic rhinitis and its impact on Asthma
AUC: Area Under the Curve
BAP: Scientific Research Projects Coordination Unit
EDN: Eosinophil-derived neurotoxin
ELISA: Enzyme-Linked ImmunoSorbent Assay
IgE: Immunoglobulin E
LMR: Lymphocyte-to-monocyte ratio
NLR: Neutrophil-to-lymphocyte ratio
ROC: Receiver Operating Characteristic
SIRI: Systemic Inflammation Response Index
SII: Systemic Immune-Inflammation Index
